# Principles or Guides for Consumptives Amenable to High Altitudes

**Published:** 1897-04

**Authors:** 


### BOOK NOTICES.

PRINCIPLES OR GUIDES FOR A BETTER SELECTION OR CLASSIFICATION OF CONSUMPTIVES AMENABLE TO HIGH ALTITUDE TREATMENT, AND TO THE SELECTION OF PATIENTS WHO MAY BE MORE SUCCESSFULLY TREATED IN THE ENVIRONMENT TO WHICH THEY WERE ACCUSTOMED PREVIOUS TO THEIR ILLNESS. By A. Edgar Tussey, M. D., Adjunct Professor of Diseases of the Chest in the Philadelphia Polyclinic and School for Graduates in Medicine. P. Blakiston, Son & Co., 1012 Walnut Street, 1896. Cloth, 144 pages, price, $1.50.

A glance at the above paragraph, which is copied entire from the title page, will sufficiently explain to the reader the aim and object of this little book. The author, in composing such a work, incidentally displays his own highly learned mind upon this important question of sending away patients who have consumption. The volume consists of a series of short, unnumbered chapters upon the conditions under which consumptives may or may not be sent to high altitudes. Each chapter considers a special division of the subject, such as "climatic vicissitudes," "residence in cities and towns" at high altitudes, "sedentary work," "hemorrhages," "muscle training," "age," "cardiac complications," etc. The limits within which one must make his prescription of change of residence for consumptives are very learnedly given, but the impression is given that they involve an amount of judgment upon the part of the reader that almost equals that of the very able author, and that they also involve an acuteness of diagnostic perceptiveness only to be found in comparatively few specialists.

The author makes some comments upon the climate of the Atlantic coast of the United States, and denies that consumption is engendered by it. He thinks "the foundation for the malady must be laid in bad habits or a deeply seated vicious inheritance." "When the vital forces are in so degenerate a condition, there is danger in any climate of some debilitating influence proving to be an exciting cause."

He gives contra-indications which should guide the medical adviser in directing the patient to stay at home. He thinks (page 73) that the occurrence of hemorrhages is not necessarily a bar to a residence in a high altitude, but points out (page 83) that it would be unwise to make the change during the continuance of a season of such hemorrhages.

In view of the amount of routine advice to make one's residence in a different climate with a higher altitude which is so constantly given, together with the number of deaths that follow upon such ill-advised change, a guide in determining what cases are suitable for locations of high altitude, and what cases may be properly directed to remain at home, is urgently needed.

This book is written to fill this need; but as was before said, it demands an increased skill upon the part of the practitioner in making an exact diagnosis of the case before him in order to give the right advice.

